# An efficient method for network traffic anomaly detection based on SHAP and deep learning

**DOI:** 10.1371/journal.pone.0356069

**Published:** 2026-08-28

**Authors:** Zhaohui Fang, Ping Xuan, Hong Ding

**Affiliations:** 1 School of Computer Science and Information Engineering, Hefei University of Technology, Hefei, China; 2 Electronic and Electrical Products Inspection Institute, Anhui Product Quality Supervision and Inspection Research Institute, Hefei, China; Posts and Telecommunications Institute of Technology, VIET NAM

## Abstract

To address the critical challenge of Denial-of-Service (DoS) attack detection in wireless sensor networks (WSNs), this study proposes an efficient anomaly detection framework that synergistically integrates SHAP (SHapley Additive exPlanations) for feature interpretation and a deep convolutional neural network (DCNN) with self-attention mechanism. The SHAP algorithm directly selects optimal feature subsets by quantifying feature contributions, eliminating the need for dimensionality reduction. Subsequently, a DCNN model enhanced with self-attention mechanisms learns spatiotemporal patterns from SHAP-refined features, improving discriminative capability for anomaly traffic. Evaluated on the UNSW-NB15 dataset, our model achieves AUROC = 0.999 and AUPRC = 0.992, surpassing state-of-the-art methods (SVM: AUROC = 0.993; XGBoost: AUPRC = 0.988). Ablation studies confirm SHAP improves MCC by 6.1% and attention mechanisms boost DoS detection precision by 3.9%. This work demonstrates that combining interpretable feature selection (SHAP) with attention-driven deep learning significantly enhances detection efficiency and accuracy, providing a viable solution for real-time WSN security.

## 1. Introduction

Wireless sensor networks (WSNs) are extensively utilized across diverse practical applications, including healthcare, commercial domains, and numerous other fields. The security of WSNs has garnered significant attention from researchers in the realm of network security [1]. WSNs execute data computation and processing, subsequently transmitting essential data to observers, thereby greatly facilitating people’s daily lives. However, if WSNs are subjected to attacks such as Denial-of-Service (DoS) attacks, it can result in catastrophic damage to WSN devices. Consequently, the effective detection of network traffic anomalies emerges as a crucial research topic.

Numerous types of network attacks currently exist, among which DoS attacks are particularly prevalent [2]. DoS attacks manifest when a server receives an overwhelming number of requests beyond its processing capacity, consequently refusing service. This mechanism inherently poses a significant challenge for detecting DoS attacks. According to recent reports by Cloudflare and NETSCOUT, the frequency and intensity of DoS and DDoS attacks have escalated significantly. In 2023 alone, Cloudflare recorded a 65% year-over-year increase in HTTP DDoS attacks, with over 7.5 million such attacks mitigated in a single quarter. Similarly, NETSCOUT’s Threat Intelligence Report highlights that attackers are increasingly targeting critical infrastructure and exploiting new vulnerabilities, with over 13 million DoS/DDoS attacks observed globally in 2023. These trends indicate the urgent need for more robust and adaptive detection methods. Recently, the number of sophisticated DoS techniques has been rapidly increasing. For instance, amplification attacks exploit vulnerabilities in Internet of Things (IoT) devices, thereby posing significant threats [3,4]. Centralized designs in Software-Defined Networking (SDN) architectures have made them prime targets for hackers, who leverage structural exploits to launch powerful DoS attacks [4]. Consequently, it is imperative to develop more effective methods for detecting network traffic anomalies.

In general, the methods for detecting abnormal network traffic primarily encompass rule-based methods, statistical-based methods, machine learning-based methods, and deep learning-based methods. Rule-based methods are straightforward to implement but necessitate frequent rule updates to address novel threats. Statistical-based methods do not require extensive labeled data but are limited in detecting emerging threats. Machine learning-based methods can handle complex patterns but demand substantial labeled data for training. Deep learning-based methods can capture intricate spatiotemporal features but require considerable computational resources and time to train the models. At this stage, machine learning-based algorithms and deep learning-based algorithms constitute two critical research directions for addressing the problem of network abnormal traffic detection.

Among these approaches, machine learning-based methods have been widely investigated for DoS attack detection because of their relatively low computational cost and interpretability. Classical algorithms, including decision tree (DT), support vector machine (SVM), and k-nearest neighbors (KNN), have demonstrated effectiveness in network anomaly detection [5]. Ma et al. [6] proposed an SVM-based algorithm for detecting network traffic anomalies, while Ahmad et al. [7] combined feature selection techniques with DT model to improve DoS detection accuracy. Alharbi et al. [8] further applied KNN for DoS attack detection. However, these methods generally rely on manually designed features and have limited capability in automatically extracting complex representations from high-dimensional traffic data.

To overcome the limitations of traditional machine learning approaches, deep learning-based methods have been introduced to automatically extract hierarchical representations from network traffic data. Convolutional neural networks (CNNs) have been widely adopted in intrusion detection because of their ability to capture local spatial correlations among traffic features. Mezina et al. [9] utilized CNNs for network attack classification and demonstrated their effectiveness in extracting discriminative traffic patterns. Patil et al. [10] proposed a PCA-based feature reduction strategy combined with a bidirectional generative adversarial network (BiGAN) for anomaly detection. Yao et al. [11] further combined PCA with CNNs for DoS traffic anomaly detection and achieved effective classification performance. Although CNN-based methods provide strong feature extraction capability, their local receptive fields limit their ability to model long-range dependencies among complex traffic features.

To further capture temporal and global dependencies in network traffic, sequential and attention-based deep learning architectures have been explored. GRU-based models have been applied in VANET anomaly detection to exploit temporal dependencies in traffic sequences [12]. Meanwhile, CNN-based real-time anomaly detection approaches have improved automatic feature extraction and reduced manual feature engineering requirements [13]. Other approaches, including clustering-based anomaly detection [14], scalable classifiers such as Mahout [15], and parallel Isolation Forest algorithms [16], have also been investigated to improve detection efficiency and adaptability. However, these methods still face challenges in balancing detection accuracy, computational cost, and adaptability to evolving attack patterns.

More recently, Transformer-based architectures have attracted increasing attention in network anomaly detection due to their self-attention mechanism and ability to capture global dependencies. Originally introduced by Vaswani et al. [17], Transformer models can dynamically assign attention weights to different features and effectively model long-range relationships without recurrent operations. Recent studies have demonstrated the effectiveness of Transformer-based approaches for intrusion detection. Transformer and large language model-based frameworks have been investigated for efficient intrusion detection systems, showing strong capability in representing complex network behaviors [18]. Time-series Transformer models have also been explored to improve anomaly detection by capturing temporal characteristics of network traffic [19]. Furthermore, hybrid architectures combining CNN and Transformer mechanisms have been proposed to integrate local feature extraction with global dependency modeling, achieving competitive performance in intrusion detection tasks [20]. For sensor networks, Transformer-based anomaly detection methods have shown potential in capturing correlations among distributed sensor nodes [21]. However, the high computational complexity of self-attention remains a challenge for deployment in resource-constrained WSN environments [21]. Furthermore, self-supervised Transformer-based frameworks have recently been proposed to improve model generalization under limited labeled data conditions, demonstrating the growing applicability of Transformer architectures to practical intrusion detection scenarios [22].

Although existing studies have significantly improved network traffic anomaly detection performance, several challenges remain. Machine learning methods still depend heavily on manually extracted features, while deep learning approaches introduce stronger representation capability at the cost of increased computational complexity. Previous studies have also explored feature selection and feature engineering techniques to improve detection performance by identifying more informative features prior to classification [23,24]. However, these approaches generally rely on conventional feature extraction strategies and provide limited interpretability regarding feature contributions. In addition, although Transformer-based models provide powerful global feature modeling ability, their computational overhead may limit deployment in resource-constrained WSN environments. Consequently, there remains a need for a lightweight yet effective framework that can jointly improve feature representation, computational efficiency, and model interpretability for practical WSN anomaly detection.

A comparative summary of representative network traffic anomaly detection methodologies is provided in Table 1. It highlights the fundamental principles, advantages, and limitations of different approaches, including traditional methods, machine learning-based methods, deep learning-based methods, and recent attention-based architectures. The comparison illustrates the evolution from conventional machine learning techniques to recent attention-based architectures, thereby motivating the development of an integrated framework suitable for resource-constrained WSN environments.

**Table 1:** Summary of network traffic anomaly detection methodologies.

Category	Representative Methods / Studies	Key Idea	Advantages	Limitations
Traditional	Rule-based	Pre-defined signature	Simple, fast,	Cannot detect novel attacks; requires
		matching	low false positives	constant matching rule updates
	Statistical-based	Modeling normal	No need for labeled	Poor detection of complex
		traffic profiles	attack data; lightweight	or evolving anomalies
Machine Learning (ML)	DT, SVM,	Learning discriminative	Handles complex patterns	Requires substantial labeled data;
	KNN [5–8]	patterns from features	better than traditional methods;	limited feature learning capacity;
			interpretable (e.g., DT)	may struggle with high-dimensional data
Deep Learning (DL)	CNN [9,11],	Automatic hierarchical	Superior feature learning;	High computational cost;
	BiGAN [10]	feature extraction from	high accuracy for	large labeled data requirement;
		raw/spatial data	known patterns	acts as a “black box”
Transformer-based Methods	Transformer architecture [17]	Self-attention based	Captures long-range dependencies;	High computational complexity
	and Transformer-based intrusion	global dependency modeling	complex traffic patterns	and memory cost
	detection methods [18–22]			
Recent Advanced Methods	Clustering-based [14]	Detecting collective deviations	Works with unlabeled data;	Less effective for novel attack
		rom normal clusters	good for known normal patterns	patterns not forming distinct clusters
	Mahout Classifier [15]	Scalable ML on	Handles large-scale	High computational resources;
		big data frameworks	data	more suited for offline analysis
		Modeling temporal dependencies	Excellent for time-series	Performance heavily reliant
	GRU-based [12]	in sequential data	traffic data; captures context	on data quality/quantity;
				complex training
	CNN for Real-time [13]	Automated feature extraction	Reduces manual feature	Requires significant computational
		for streaming data	engineering; high accuracy	power for real-time processing
	Parallel Isolation	Efficient anomaly scoring	Fast training and	Sensitive to data distribution assumptions;
	Forest [16]	via parallel tree ensembles	inference; scalable	may have higher false positives

In this paper, we present a more effective method for the research of network traffic anomaly detection. Unlike traditional methods that often rely on reducing feature dimensionality or require extensive manual feature engineering, we first employ a novel feature selection method, namely SHAP [25,26], to directly extract important features from the original dataset. This method not only characterizes feature importance but also evaluates feature behavior, offering superior feature extraction capability. Furthermore, we construct a deep CNN model integrated with a self-attention mechanism for detecting DoS attacks in WSNs. Unlike conventional deep learning models that may lose feature information through pooling layers, our model replaces the pooling layer with an attention mechanism. This allows it to capture crucial feature information during the learning process, thereby enhancing the model’s ability to classify network anomaly traffic effectively. Our proposed model demonstrates a good detection accuracy by learning the traffic characteristics after feature selection using SHAP.

### 1.1. Article structure

This article is organized into five main sections to provide a logical flow from problem identification to solution validation and future outlook. Section 1 (Introduction) establishes the research context, reviews related work, and highlights the motivation and core contributions of this study. Section 2 (Model and Methodology) details the proposed framework, encompassing SHAP-based interpretable feature selection, the DCNN architecture integrated with a self-attention mechanism, and the comprehensive training protocol. Section 3 (Experiments and Results) presents the experimental setup, benchmark dataset, evaluation metrics, and a thorough analysis of results, including comparative studies, ablation experiments, and statistical tests. Section 4 (Future Research Directions) outlines potential avenues for extending this work. Finally, Section 5 (Conclusions) summarizes the key findings and discusses the broader implications and remaining challenges.

### 1.2. Contributions

The primary contributions of this research are threefold:

1. We introduce a novel feature selection method, SHAP, which directly extracts important features from the data rather than reducing feature dimensionality. This method provides a more accurate and interpretable way to identify critical features for anomaly detection.
2. We develop a deep CNN model with a self-attention mechanism tailored for DoS attack detection in WSNs. The attention mechanism enables the model to focus on key features, improving its ability to distinguish between normal and abnormal network traffic compared to traditional deep learning models.
3. Through numerical experiments on the UNSW-NB15 dataset [27], we demonstrate that our proposed model outperforms other methods, such as SVM and XGBoost, across six classification evaluation metrics: Recall, Precision, F1-score, Accuracy, Specificity, and Matthews correlation coefficient (MCC). These results highlight the effectiveness of our approach in network traffic anomaly detection.

### 1.3. Limitations and challenges

Despite its promising performance, the proposed framework faces limitations and practical deployment challenges:

1. Computational and Deployment Constraints: The integration of the self-attention mechanism increases model complexity. While beneficial for accuracy, this can lead to higher computational overhead and memory footprint, posing a challenge for real-time inference on resource-constrained WSN nodes. Balancing high detection performance with the stringent efficiency requirements of edge devices remains a key challenge.
2. Generalization to Evolving Threats: The model’s effectiveness is validated on existing benchmark data. Its ability to generalize to novel, unseen attack patterns or significantly different network environments without performance degradation is uncertain. Continual model updates are required, which increases maintenance costs. Developing adaptive mechanisms for evolving threats is a significant research challenge.
3. Interpretability-Practicality Balance: While SHAP provides deep, instance-level model interpretability, the complexity of its outputs (e.g., detailed force plots) may hinder direct practical utility for network operators who need clear, actionable insights. Translating complex model explanations into simple, operational decision-support tools presents a practical challenge.

## 2. Model and methodology

**Fig 1:**
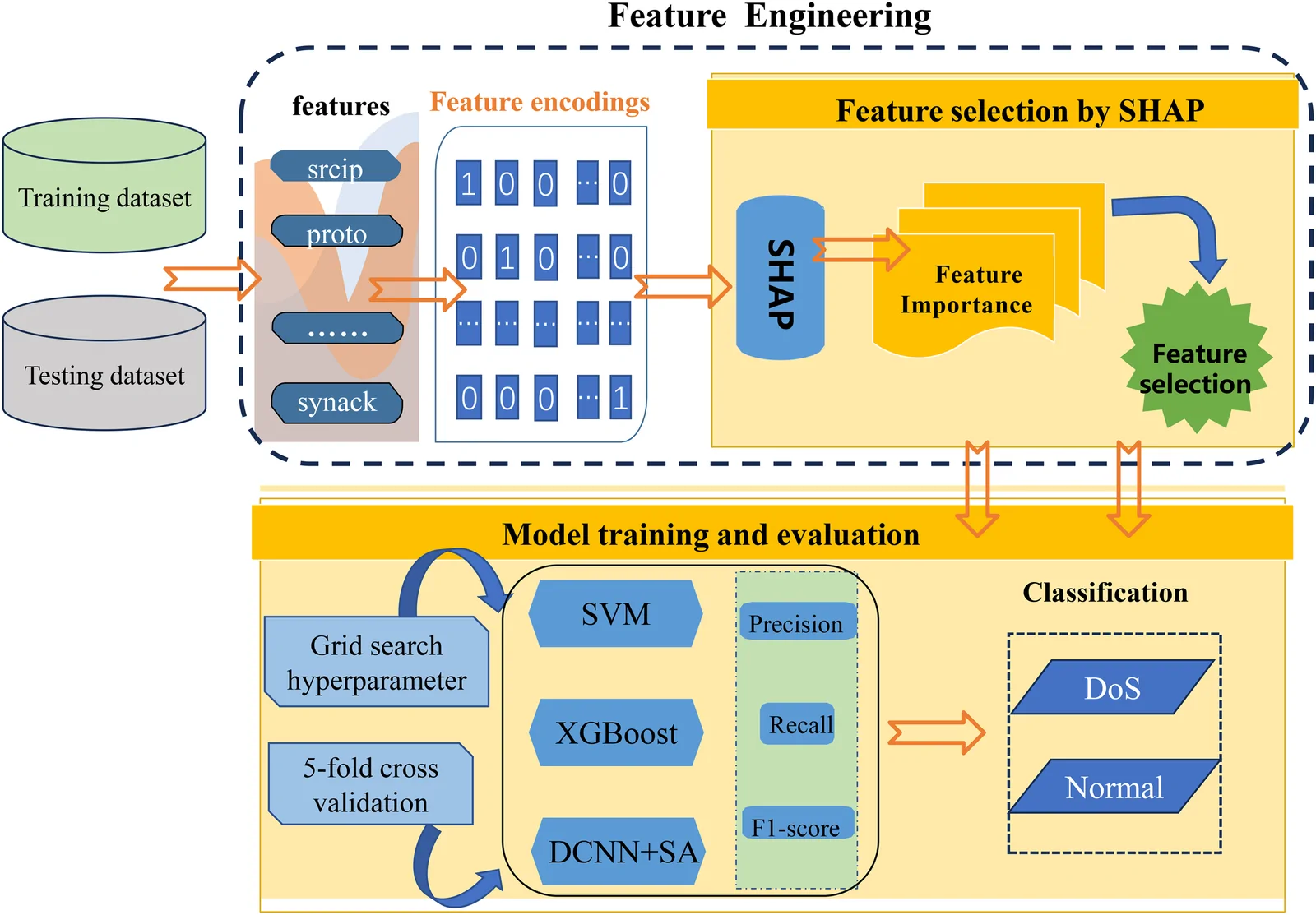
The overall framework of our proposed SHAP-DCNN+SA model for network traffic anomaly detection.

This section introduces the core framework proposed for addressing DoS attack detection in wireless sensor networks (WSNs), which synergistically integrates two key components: SHAP (SHapley Additive exPlanations)-driven interpretable feature selection and a deep convolutional neural network (DCNN) augmented with a self-attention (SA) mechanism. As illustrated in Fig 1, the framework is structured into two interdependent modules Feature Engineering (feature encoding + SHAP-based feature selection) and Model Training & Evaluation (DCNN+SA training and performance validation). Its key design goals are tailored to WSNs practical needs: improving feature interpretability (to clarify critical DoS-related features), reducing redundant information (to lower resource consumption in WSNs), and enhancing anomaly discrimination (to boost the accuracy of distinguishing DoS attacks from normal traffic). Fig 1 illustrates the overall framework of our proposed SHAP-DCNN+SA model for network traffic anomaly detection.

### 2.1. Feature selection by SHAP and data preprocessing

Feature selection plays a crucial role in feature engineering, especially for extracting important features from high-dimensional complex datasets. SHAP is a game-theoretic approach that provides a unified measure of feature importance by calculating the Shapley values, which quantify the contribution of each feature to the model’s predictions [28]. This method is particularly powerful because it not only characterizes feature importance but also evaluates feature behavior. Unlike other feature selection methods such as PCA, which reduces dimensionality by extracting variance information, SHAP directly selects the optimal feature subsets, ensuring better feature learning capacity and detection accuracy.

Before applying SHAP for feature selection, a comprehensive data preprocessing pipeline was implemented to ensure the integrity and suitability of the data for analysis. The specific steps and techniques are detailed below:

Data Cleaning: Missing values were handled using a two-pronged approach. For numerical features with less than 5% missingness (e.g., packet_size, flow_duration), missing values were imputed using the median value of the feature due to its robustness to outliers. For numerical features with higher missing rates (>5%) or categorical features (e.g., protocol_type, service), missing values were treated as a separate category (’Unknown’). This strategy preserves the potential information about missingness, which might be indicative of certain attack patterns, while avoiding the introduction of spurious relationships that could arise from imputing categorical or highly sparse numerical features with a central tendency measure. Outliers were identified using the Interquartile Range (IQR) method, where data points below Q1-1.5*IQR or above Q3 + 1.5*IQR (Q1 and Q3 being the 25th and 75th percentiles, respectively) were considered outliers. These outliers were then Winsorized (capped) at the 1st and 99th percentiles to mitigate their undue influence on the model without losing valuable data points or introducing excessive bias.

Normalization: All numerical features were scaled to a common range of [0,1] using Min-Max normalization, implemented with the MinMaxScaler from the Python scikit-learn library (v1.2+). The scaler was fitted exclusively on the training data, and the resulting transformation parameters (i.e., min(*x*) and max(*x*) for each feature) were then applied to transform both the training and test sets. The formula for each feature value *x* was: $x_{norm}=\frac{x-min(x)}{max(x)-min(x)}$. This step is critical prior to SHAP analysis as the calculated Shapley values are sensitive to the scale of the input features. Normalization ensures that the Shapley values are comparable across all features, allowing for a fair and consistent assessment of their relative importance. The use of this established library ensures the reproducibility and reliability of the normalization process.

Encoding: Categorical variables (e.g., ’protocol_type’, ’service’, ’state’) were converted into numerical formats using one-hot encoding. This method was chosen over label encoding or ordinal encoding to avoid introducing any spurious ordinal relationships or implying incorrect magnitudes between categories that do not inherently exist (e.g., implying TCP > UDP), which could mislead the subsequent interpretation of feature importance by SHAP.

Data Splitting: The preprocessed dataset was stratified and partitioned into training (70%) and testing (30%) sets to maintain the original class distribution in both subsets, crucial for handling the inherent imbalance. All subsequent steps, including SHAP analysis and feature selection, were conducted exclusively on the training set to prevent any information leakage from the test set, ensuring an unbiased evaluation of the model’s generalization performance. The tree-based LightGBM classifier was first trained on this preprocessed training data. Subsequently, the SHAP TreeExplainer was employed on this trained LightGBM model to calculate the Shapley values for each instance in the training set. It is critical to note that the SHAP analysis and the subsequent calculation of global feature importance (mean |SHAP value|) were performed exclusively on the training set. This strict separation prevents any information from the test set from leaking into the feature selection process, which could otherwise lead to optimistically biased performance estimates and compromise the model’s generalizability.

The SHAP method calculates the Shapley values for each feature and each instance, representing the average marginal contribution of each feature across all possible coalitions of features. To determine global feature importance, the mean absolute Shapley value (mean |SHAP value|) for each feature across the entire training dataset was computed. Features with higher mean |SHAP value| are considered more important for the model’s overall predictions. While computing SHAP values via cross-validation on the entire training set could offer a more robust estimate of feature importance, we prioritized computational efficiency and the stability of the TreeExplainer with a fixed model for this study. Our approach of using a single, held-out training set for SHAP calculation is a widely adopted and methodologically sound practice that effectively mitigates bias.

**Fig 2:**
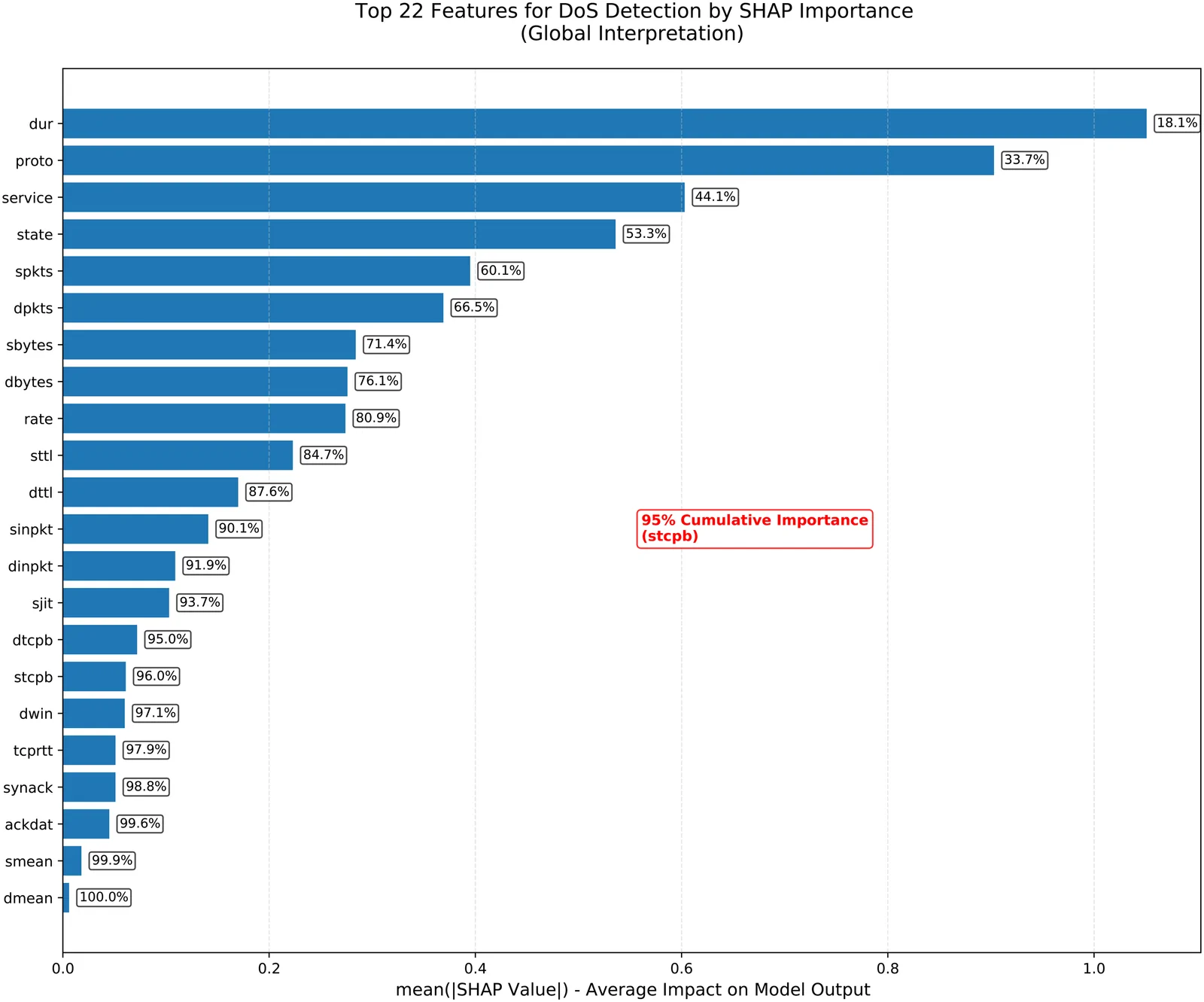
Global feature importance based on mean absolute SHAP values. The top 22 features selected for the final model are shown, ranked by their impact on the prediction output. The cumulative importance percentage is annotated on each bar. The threshold for 95% cumulative importance.

**Fig 3:**
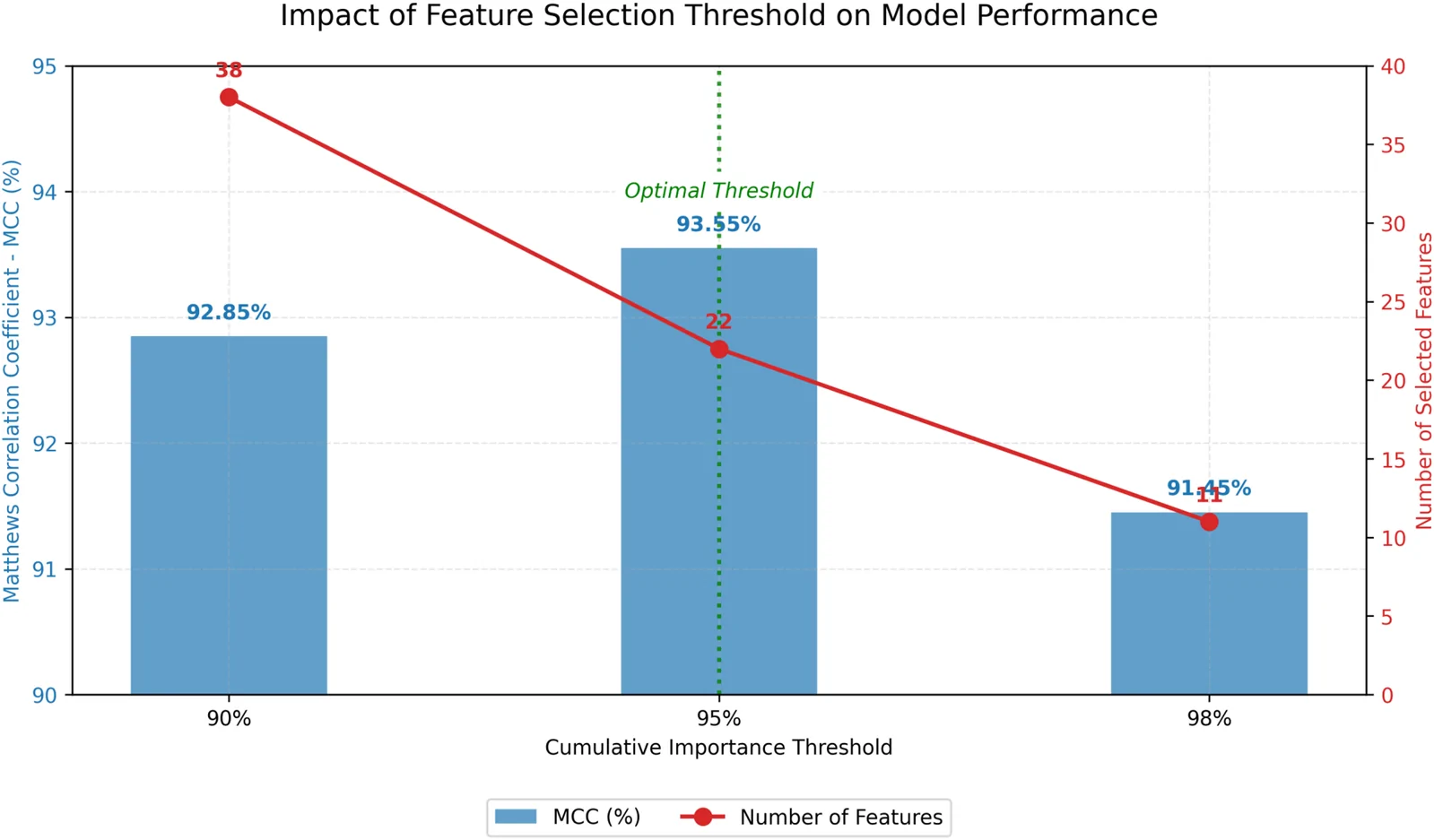
Impact of feature selection threshold on model performance.

Beyond global feature importance, SHAP provides deep model interpretability by revealing how each feature contributes to individual predictions across different attack scenarios. For instance, while Packet Size might be globally important, its specific contribution varies: exceptionally large packets might strongly indicate a bandwidth exhaustion attack in one scenario, while in another context, a specific Protocol Type (e.g., ICMP) combined with a short Flow Duration might be the primary contributor to identifying a flood attack. This instance-level explanation capability allows us to understand the model’s decision boundaries not as fixed lines in a high-dimensional space but as dynamic boundaries influenced by the cooperative or antagonistic contributions of features specific to each network traffic instance. To effectively illustrate how features influence both individual predictions and the overall model decision process, we include visualizations such as SHAP summary plots and force plots (see Figs 2 and 3). Analyzing these SHAP force plots for misclassified instances can further reveal scenarios where the model’s decision boundary might be suboptimal, providing crucial insights for model refinement.

#### Exact criteria for feature selection based on SHAP values.

The specific criterion for selecting the final feature subset was based on cumulative feature importance. Features were first sorted in descending order of their mean |SHAP value|. The cumulative sum of these mean |SHAP value| was then calculated. The feature importance threshold was defined as the minimum mean |SHAP value| required for the cumulative importance of the selected feature subset to reach or exceed 95% of the total cumulative importance from all features. Features with a mean |SHAP value| greater than or equal to this threshold were retained. This criterion ensures that the selected subset captures the vast majority (95%) of the total explanatory power (as measured by SHAP) while effectively discarding features with negligible contributions, thereby reducing dimensionality and potential noise. To visually interpret the results of our SHAP analysis and justify the feature selection threshold, we generated a summary plot (Fig 2) and a dedicated cumulative importance plot (Fig 3). The summary plot ranks the top features by their mean absolute SHAP value, confirming the global importance of key features (e.g., packet_size, flow_duration) and providing insights into the distribution of their impacts. The cumulative importance plot (Fig 3) explicitly visualizes the relationship between the number of selected features and the total explanatory power captured. This plot clearly shows that the 95% threshold represents a critical inflection point where adding more features yields diminishing returns in importance gain. This choice effectively balances model performance and complexity: it retains the most predictive features to maintain high detection accuracy (as quantified by the sensitivity analysis in Section 2.2) while discarding a substantial number of low-contributing features (reducing the feature set from the original 47–22). This reduction directly decreases computational overhead and model size, enhancing the potential for deployment in resource-constrained environments like WSNs, without sacrificing predictive capability. The annotations in both figures collectively provide a transparent and visual justification for the selected threshold.

For DoS attack detection in our study, applying this criterion with the 95% cumulative importance threshold identified the following top-ranked critical features (among others):

Packet Size: Larger packet sizes can indicate potential DoS attacks as they may overwhelm the network bandwidth or processing resources. Flow Duration: Abnormal durations of network flows (either too short or excessively long) can signal DoS activities, such as flash crowds or slow-rate attacks. Protocol Types (one-hot encoded features): Certain protocols (e.g., ICMP for amplification attacks) may be more susceptible to or indicative of specific DoS attacks. Source and Destination IP Addresses(or their derived features like transaction counts): Frequent connection requests from a single source IP address or to a specific destination IP/port can indicate DoS attempts like SYN floods or HTTP floods.

These features were selected because their high mean absolute SHAP values significantly contributed to the cumulative importance, indicating they provide critical insights into the behavior distinguishing normal traffic from DoS attacks. Furthermore, their consistent and significant contributions across various individual predictions, as observed in the SHAP summary plots and dependence plots, reinforce their role in shaping the model’s decision boundaries across diverse attack patterns.

### 2.2. Sensitivity analysis of feature importance threshold and its impact

In our study, to select the most impactful features and reduce potential noise, a threshold for feature importance was set based on the mean absolute Shapley values. The methodology for determining this threshold involved an analysis of the cumulative feature importance, as described in Section 2.1. While the 95% cumulative importance threshold was initially chosen, a sensitivity analysis was rigorously performed to justify this specific choice. The results of this analysis are summarized in Fig 3 and Table 2.

**Table 2:** Results of sensitivity analysis on feature importance thresholds.

Cumulative Importance Threshold	Number of Features Selected	Matthews Correlation Coefficient (MCC)
90%	38	92.85%
95%	22	93.55%
98%	11	91.45%

#### Sensitivity analysis methodology.

We compared the performance of our final DCNN+SA model trained on three different feature subsets selected using thresholds corresponding to 90%, 95%, and 98% of the total cumulative SHAP importance. For each threshold:

The subset of features whose cumulative mean absolute SHAP value reached the specified percentage (90%, 95%, 98%) was identified. The DCNN+SA model was trained from scratch (including hyperparameter tuning specific to that feature subset if necessary) using only these selected features. Model performance was evaluated using 5-fold cross-validation on the training set to ensure robustness and avoid overfitting to a single split.

The Matthews Correlation Coefficient (MCC) was used as the primary evaluation metric for this sensitivity analysis due to its sensitivity to class imbalance and its balanced nature, providing a single informative measure across all confusion matrix categories.

#### Results of sensitivity analysis.

The results (Fig 3 and Table 2) clearly indicated that the choice of threshold significantly impacted model performance, particularly in terms of its effect on false positives and false negatives for the critical DoS attack class:

Higher Threshold (98% Cumulative Importance): This aggressive feature reduction led to a noticeable drop in MCC (approximately 2.1%), primarily attributable to a sharp increase in false negatives (missed DoS attacks). The loss of moderately important features degraded the model’s ability to capture the full spectrum of attack signatures, thereby compromising the overall attack detection capability and recall for the minority class.

Lower Threshold (90% Cumulative Importance): Retaining a larger set of low-importance features slightly degraded the MCC (approximately 0.7%) and led to a noticeable increase in false positives (false alarms). The model, burdened by noisy and redundant features, learned less discriminative patterns, reducing its precision in distinguishing normal traffic from actual attacks.

Intermediate Threshold (95% Cumulative Importance): This threshold achieved the optimal balance, yielding the highest MCC score. It effectively retained the features necessary to minimize both false negatives and false positives. This optimal trade-off resulted in the most robust attack detection capability, maintaining high recall (minimizing missed attacks) while achieving high precision (minimizing false alarms), which is crucial for reliable security monitoring in WSNs where both oversight and false alerts carry significant costs.

#### Conclusion from sensitivity analysis.

Therefore, based on this empirical sensitivity analysis, the 95% cumulative importance threshold was rigorously justified and selected for our final model. This threshold provides the best operational trade-off by optimizing the balance between false positives and false negatives, as reflected in the maximized MCC. It ensures high detection accuracy and reliability for the critical DoS attack class while maintaining a favorable balance between model complexity, computational efficiency, and interpretability. This analysis underscores the significant impact of the feature importance threshold selection on the final model’s performance, generalizability, and practical utility for security applications.

### 2.3. Classifier

In this subsection, we introduce three significant classifiers employed in the numerical experiments for network traffic anomaly detection. The classification models comprise two machine learning-based models, namely support vector machine (SVM) and XGBoost, and a deep learning model, deep convolutional neural network (DCNN). Specifically, SVM is a type of supervised learning model that effectively performs binary classification on datasets [29]. Abusitta et al. [30] demonstrated that the SVM model effectively detects DoS attacks, proving to be highly efficient for identifying network traffic anomalies. XGBoost, a machine learning model that has demonstrated outstanding performance in network intrusion detection tasks [31,32], has recently gained prominence. Liu et al. [33] developed a novel method, m5Cpred-XS, integrating SHAP and XGBoost for predicting m5C sites. Experimental results revealed that XGBoost performs exceptionally well in processing sparse and high-dimensional data. CNN, known for its feature extraction capabilities, has proven effective in target recognition, text classification, and anomaly detection [34]. Generally, the architecture of CNN incorporates three primary layers: convolution, pooling, and fully connected layers.

**Fig 4:**
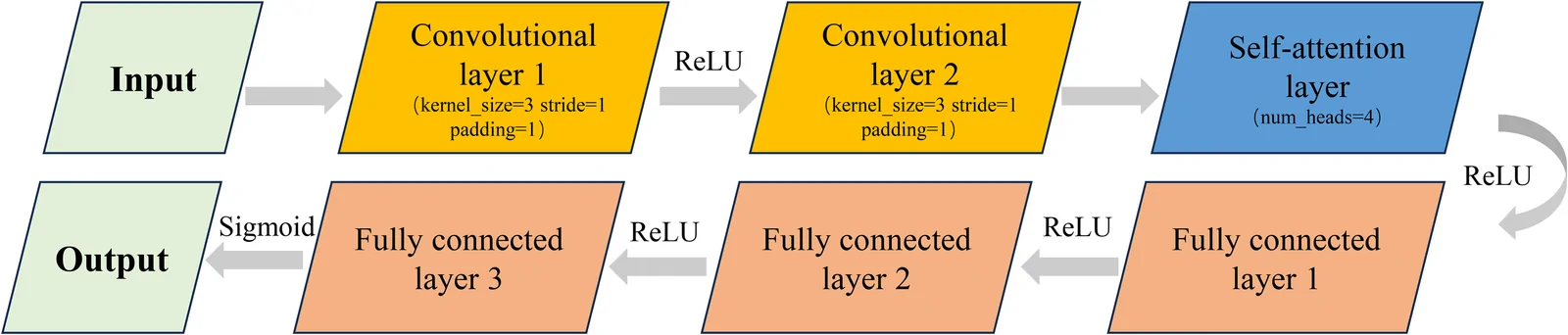
The proposed DCNN model.

In this study, we construct a deep CNN model for detecting DoS attacks, incorporating an attention mechanism to replace the traditional pooling layer to enhance important feature extraction. Fig 4 depicts the 6-layer deep convolutional structure utilized in this research, which comprises two convolution layers, one attention mechanism layer, and three fully connected layers. The first convolutional layer uses 3×3 kernels with a stride of 1 and padding of 1, while the second convolutional layer maintains the same kernel size, stride, and padding configuration. In the experiments, our proposed DCNN model demonstrates robust feature extraction capabilities, achieving good classification accuracy for traffic anomaly detection.

### 2.4. Attention mechanism

The attention mechanism can help models assign different weights to each part of the input features, extract more critical and important information, and make more accurate judgments. However, the integration of self-attention mechanisms may introduce additional computational overhead, which could impact inference time and model size. This is particularly relevant in resource-constrained environments such as wireless sensor networks (WSNs), where real-time deployment requires a balance between model performance and computational efficiency [35]. Future work should further investigate the trade-offs between the improved feature learning capabilities provided by attention mechanisms and their associated computational costs, especially in the context of real-time anomaly detection on edge devices with limited resources. In our model, by introducing the self-attention (SA) mechanism, it can avoid feature loss caused by pooling downsampling and improve the model’s feature learning ability. This is particularly beneficial for network traffic anomaly detection, as it allows the model to focus on the most relevant features that are indicative of DoS attacks. By capturing the temporal dependencies and hierarchical relationships among features, the attention mechanism enhances the model’s ability to distinguish between normal and abnormal traffic patterns. In practice, the attention mechanism mainly includes two processes: one is to calculate the weight coefficient based on query (𝐐) and key (𝐊); the second is to weight and sum the value (𝐕) based on the weight coefficient. The mathematical description of attention formula is as follows:


$$\begin{array}{c} Attention(𝐐,𝐊,𝐕)=softmax\left(\frac{𝐐{𝐊}^{T}}{\sqrt{d_{k}}}\right)𝐕, \end{array}$$ (1)

where 𝐐,𝐊, and 𝐕 are the query, key and value matrix, respectively. And $d_{k}$ is the dimension of 𝐊.

This mechanism allows the model to dynamically adjust its focus on different features, thereby improving the accuracy and robustness of anomaly detection.

### 2.5. Model hyperparameter settings and training protocol

To address the significant class imbalance in the dataset (detailed in Section 3.1) during model training, we employed a dual strategy beyond the selection of appropriate evaluation metrics. The Synthetic Minority Over-sampling Technique (SMOTE) was applied exclusively to the training folds to generate synthetic samples for the minority DoS class, balancing the class distribution. Concurrently, class-weighted loss functions were implemented, assigning a higher penalty to misclassifications of the minority DoS attack samples based on the inverse class frequency. This combined approach compels the model to focus more on learning the characteristics of the under-represented attacks during the optimization process.

In this study, we constructed a deep convolutional neural network (DCNN) enhanced with a self-attention mechanism (DCNN+SA). The model architecture consists of six layers: two convolutional layers, one self-attention layer, and three fully connected layers. The first convolutional layer uses a 3×3 kernel, stride of 1, padding of 1, and ReLU activation, outputting 64 feature maps. The second convolutional layer has the same kernel size, stride, and padding. The self-attention layer employs 4 attention heads. The subsequent fully connected layers contain 128, 64, and 1 units, respectively, with ReLU activation for the first two and sigmoid activation for the final layer.

The hyperparameter optimization for the DCNN+SA model was conducted using an exhaustive grid search over the predefined search space. A stratified 5-fold cross-validation was employed on the training set (70% of the total data) for hyperparameter tuning (the inner loop of the nested CV). This grid search involved evaluating a total of 3×3×3×3×3×2×2×3=2916 unique hyperparameter combinations to identify the optimal configuration. The model selection criterion was based on the highest average Matthews Correlation Coefficient (MCC) across the 5 validation folds, as it provides a balanced measure for imbalanced datasets. This inner CV process inherently helps prevent overfitting to a specific validation split during hyperparameter optimization. For the baseline models (SVM, RF, XGBoost), a similar grid search methodology with stratified 5-fold CV was applied, ensuring a consistent and fair tuning process across all compared models.

The final hyperparameter configuration, determined through this process, is as follows: learning rate = 0.0005, batch size = 128, attention heads = 4, maximum number of epochs = 50, L2 regularization (weight decay) = 0.001, optimizer = Adam (with $\beta_{1}=0.9$, $\beta_{2}=0.999$), and early stopping patience = 10 epochs. The loss function was binary cross-entropy, a fixed random seed of 42 was systematically applied across all relevant components-including Python, NumPy, TensorFlow, and scikit-learn-to guarantee consistent initialization and data shuffling, thereby ensuring complete reproducibility of all experimental results. All experiments were conducted on a standardized hardware configuration featuring an NVIDIA RTX 3080 GPU and Intel Core i7-11700K processor, with software environments including Python 3.8.13, TensorFlow 2.9.1, and scikit-learn 1.2.0, ensuring no hardware-induced variations affected the reported results. A learning rate scheduler (ReduceLROnPlateau) was also employed, which reduced the learning rate by a factor of 0.1 if the validation loss plateaued for 5 consecutive epochs.

Training was conducted with a maximum of 50 epochs, and early stopping was applied to halt training if the validation loss did not improve for 10 consecutive epochs, which served as an effective measure to prevent overfitting during the training process on each fold. The model typically converged within 30–40 epochs. The complete set of hyperparameters, including those for the baseline models, is comprehensively summarized in Table 3 for full transparency.

**Table 3:** Comparative performance of different models on the UNSW-NB15 dataset.

Method	Class	Recall	Precision	F1-score	Accuracy	Specificity	MCC
SVM-SHAP	Normal	98.32%	99.26%	98.78%	97.82%	93.32%	88.38%
	DoS	93.32%	85.98%	89.50%			
XGBoost-SHAP	Normal	98.91%	99.71%	99.31%	98.76%	97.38%	93.36%
	DoS	97.38%	90.81%	93.98%			
GRU-SHAP	Normal	93.76%	99.24%	96.42%	93.74%	93.76%	73.36%
	DoS	93.52%	62.36%	74.83%			
CNN-GRU-SHAP	Normal	95.01%	99.59%	97.24%	95.15%	95.01%	78.69%
	DoS	96.45%	68.11%	79.84%			
CNN-Transformer-SHAP	Normal	93.60%	99.23%	96.33%	93.58%	93.60%	72.84%
	DoS	93.42%	61.73%	74.34%			
DCNN+SA-SHAP	Normal	99.04%	99.63%	99.34%	98.81%	96.70%	93.55%
	DoS	96.70%	91.78%	94.18%			
RF	Normal	98.65%	99.42%	99.03%	98.23%	95.12%	90.45%
	DoS	95.12%	88.67%	91.78%			

### 2.6. Complexity and deployment considerations

A comprehensive complexity analysis is crucial for understanding the practical applicability of the proposed DCNN+SA model, especially in resource-constrained environments like WSNs. This analysis encompasses computational costs during training and inference, model size, and the inherent trade-offs involved in deployment.

Training Time: The training complexity of the DCNN+SA model is primarily influenced by the number of parameters, the size of the training data, and the number of epochs. Our model, with its convolutional and self-attention layers, requires more computational resources for training compared to traditional machine learning models like SVM or XGBoost. The hyperparameter tuning process, involving grid and random search, further adds to the offline training overhead. However, this is a one-time cost incurred on a powerful server or cloud platform, which is acceptable given the performance gains.

Inference Time and Model Size: The inference time is a critical metric for real-time anomaly detection. The self-attention mechanism enhances feature learning by modeling global feature dependencies; however, it introduces additional computational overhead compared with conventional convolutional operations due to the computation of query, key, and value matrices (Q, K, V) and the corresponding attention weights. Compared with lightweight machine learning models such as SVM, Random Forest, and XGBoost, the proposed SHAP-DCNN+SA framework requires higher computational resources during inference but provides substantially improved feature representation and detection performance, as demonstrated by the higher MCC and F1-score reported in Sections 3.3 and 3.7. In contrast, more sophisticated deep learning architectures, including GRU, CNN-GRU, and CNN-Transformer, generally incur even greater computational complexity because of sequential computation or attention-intensive structures. However, the experimental results show that these architectures do not outperform the proposed SHAP-DCNN+SA framework on the UNSW-NB15 dataset, indicating that increased computational complexity does not necessarily translate into superior detection performance. Therefore, the proposed framework provides a more favorable balance between detection accuracy and computational cost for practical WSN anomaly detection. The final model size, determined by the number of trained parameters (weights and biases), is approximately 0.33 MB (82,625 parameters, stored as 32-bit floats). Although this model size remains non-negligible for highly resource-constrained sensor nodes, optimization strategies such as model compression, pruning, quantization, and edge-cloud collaborative inference can further reduce deployment overhead while preserving the high detection performance achieved by the proposed framework.

Deployment Trade-offs in Resource-Limited Settings: Deploying the DCNN+SA-SHAP system in real-world WSNs involves significant trade-offs between detection accuracy and resource consumption (computational power, memory, and energy). The high accuracy and robust performance of our model come at the cost of increased computational demand, which may challenge typical sensor nodes with limited processing capabilities (e.g., micro-controllers). To mitigate this, several strategies can be employed: (1) Model Optimization: Techniques such as quantization (reducing numerical precision of parameters), pruning (removing redundant parameters), and knowledge distillation (training a smaller, faster model) could be explored to reduce the model size and accelerate inference without severely compromising performance. (2) Edge-Cloud Collaboration: Offloading complex model inference to a more powerful edge gateway or a cloud server could be a viable architecture, where sensor nodes primarily perform data collection and preprocessing. However, this introduces dependencies on network connectivity and latency. (3) Hardware Acceleration: Utilizing specialized hardware like Tensor Processing Units (TPUs) or Neural Processing Units (NPUs) at the edge gateways could significantly enhance inference speed and energy efficiency. The choice among these strategies depends on the specific constraints and requirements of the target application, highlighting the need for a careful cost-benefit analysis when deploying advanced deep learning models in practical, resource-scarce scenarios.

## 3. Experiments and results

In this section, we present numerical simulations to evaluate the effectiveness of the proposed SHAP-DCNN+SA framework for network traffic anomaly detection. To provide a comprehensive performance evaluation, our model is compared with representative machine learning models (SVM, Random Forest(RF), and XGBoost) as well as representative deep learning architectures, including GRU, CNN-GRU, and CNN-Transformer models, on the same benchmark dataset.

### 3.1. Benchmark dataset

We use the public dataset UNSW-NB15 [27] for simulations. This dataset is a widely recognized benchmark for network intrusion detection systems, containing a diverse set of network traffic data, including normal traffic and various types of attacks. It comprises 47 feature types and nine attack types, with the data derived from real-world network traffic. In our study, we focus on detecting DoS attacks in WSNs, where the dataset includes 92,998 positive samples (normal traffic) and 16,353 negative samples (DoS attacks). To ensure the reproducibility of our experiments, which is a key criterion for scientific validation, we have made our code and the specific data splits (training and testing sets) used in this study publicly available. The dataset was split into training and testing sets at a ratio of approximately 60% for training and 40% for testing, ensuring the model is trained on a sufficiently large dataset and tested on a substantial independent dataset to evaluate its generalization performance. The selection of UNSW-NB15 is based on its comprehensive coverage of network traffic types and attack categories, making it suitable for our research.

Before using the dataset, several preprocessing steps were applied. Data cleaning involved imputing missing values and handling outliers. Normalization scaled numerical features to a [0,1] range, and categorical variables were encoded using one-hot encoding. The SHAP method was then used for feature selection to identify the most important features for DoS detection, reducing dimensionality while retaining critical information.

The dataset exhibits a significant class imbalance, with normal traffic samples (92,998) substantially outnumbering DoS attack samples (16,353). This imbalance could bias the model towards the majority class, potentially leading to suboptimal detection performance for DoS attacks. The specific techniques employed to mitigate this issue during training are detailed in Section 2.5. The overall strong performance of our model, particularly in terms of Recall and Precision for the DoS class as presented in Section 3.3 and evidenced by the number of correctly identified DoS attacks in the confusion matrices (Section 3.5), demonstrates the effectiveness of our overall approach in handling class imbalance.

### 3.2. Evaluation criteria

In the experiments, we utilize a comprehensive set of evaluation metrics to thoroughly assess model performance, with particular emphasis on measures that are sensitive to class imbalance. While the Area Under the Receiver Operating Characteristic Curve (AUROC) and the Area Under the Precision-Recall Curve (AUPRC) provide valuable insights into model performance, relying solely on them can be misleading for highly imbalanced datasets like UNSW-NB15. AUROC may present an overly optimistic view due to the dominance of the majority class (normal traffic), while AUPRC, though more focused on the minority class, still offers a summarized view. Therefore, to mitigate potential biases and ensure a comprehensive and robust evaluation, we complement these metrics with a suite of additional performance indicators. These include Recall, Precision, F1-score, Accuracy (ACC), Specificity (SP), and the Matthews correlation coefficient (MCC). Furthermore, we report class-specific metrics, such as Recall, Precision, and F1-score for both the Normal and DoS attack categories, as these provide crucial insights into the model’s performance on each individual class, which is paramount for imbalanced datasets. Finally, confusion matrices are employed to offer a granular view of classification errors. The detailed rationale for selecting this comprehensive set of metrics is as follows.

AUROC reflects the model’s ability to distinguish between positive and negative samples across all classification thresholds. While valuable for assessing overall ranking capability, its interpretation can be optimistic under severe imbalance, as the large number of true negatives (normal traffic) can inflate the score. Nevertheless, a high AUROC value remains indicative of a model’s good separable capacity and is included for comparison with prior studies.

AUPRC, in contrast, focuses exclusively on the performance regarding the positive (minority) class-DoS attacks in our case. It evaluates the trade-off between Precision and Recall, making it a more informative and reliable metric than AUROC for imbalanced scenarios where the primary interest lies in correctly identifying the rare anomalies. A high AUPRC value signifies that the model achieves both high detection rates (Recall) and high reliability of those detections (Precision) for the minority class, which is the central challenge in anomaly detection.

The F1-score, defined as the harmonic mean of Precision and Recall, provides a single metric that balances the concerns of both false positives and false negatives for a specific class. Reporting the F1-score separately for the Normal and DoS classes allows for a direct comparison of how well the model performs on the majority versus the minority class. A significant disparity between these per-class F1-scores would highlight a model’s bias, whereas similar, high scores indicate robust performance across classes.

The Matthews Correlation Coefficient (MCC) produces a high score only if the prediction achieved good results in all four confusion matrix categories (TP, TN, FP, FN), relative to the size of the dataset. It is generally regarded as a balanced measure, even when the classes are of very different sizes, making it exceptionally well-suited for evaluating performance on our imbalanced dataset.

Finally, confusion matrices are employed to provide a granular view of model performance across different classes. They intuitively display the counts of true positives, false positives, true negatives, and false negatives, enabling a detailed analysis of the specific types of errors made by the model (e.g., missed attacks vs. false alarms). This level of detail is essential for understanding the practical implications of the model’s performance and for guiding further refinements to the anomaly detection system. The formulas of the evaluation metrics are defined as follows.


$$\begin{array}{c} Recall=\frac{TP}{FN+TP}, \end{array}$$ (2)

$$\begin{array}{c} Precision=\frac{TP}{TP+FP}, \end{array}$$ (3)

$$\begin{array}{c} F1-score=\frac{2\times Precision\times Recall}{Precision+Recall}, \end{array}$$ (4)

$$\begin{array}{c} Accuracy=\frac{TN+TP}{TN+FP+FN+TP}, \end{array}$$ (5)

$$\begin{array}{c} Specificity=\frac{TN}{TN+FP}, \end{array}$$ (6)

$$\begin{array}{c} MCC=\frac{TN\times TP-FN\times FP}{\sqrt{\left(TN+FP)(FN+TP)(TN+FN)(TP+FP\right)}}, \end{array}$$ (7)

where *TP*, *TN*, *FP* and *FN* represent the number of true positive, true negative, false positive and false negative samples between model prediction and the target sample’s true category, respectively.

### 3.3. Models based on feature selected by SHAP

To provide an unbiased estimate of the generalization performance of our final proposed model (DCNN+SA with SHAP-selected features and the optimal hyperparameters from Section 2.5), we employed an outer loop of stratified 5-fold cross-validation on the entire dataset. This constitutes the outer loop of the nested cross-validation framework.

In each iteration of the outer loop: The data was split into a training fold (80%) and a held-out test fold (20%), preserving the class distribution (stratified).

On the training fold, the entire process was repeated: SHAP analysis and Feature Selection were performed de novo, and the DCNN+SA model was trained using the same hyperparameter optimization procedure described in Section 2.5 (i.e., an inner 5-fold CV on this training fold) to find the best hyperparameters for this specific data split.

The final model, refit on the entire training fold using these best hyperparameters and the selected features, was then evaluated on the completely unseen test fold.

The performance metrics reported in Table 4 (Recall, Precision, F1-score, Accuracy, Specificity, MCC) represent the average (standard deviation) of the results across all five test folds of the outer cross-validation. This rigorous evaluation protocol ensures that the reported performance is a robust estimate of the model’s ability to generalize to unseen data and provides strong evidence against overfitting, as the model is evaluated on multiple independent test sets that were not involved in any part of the feature selection, hyperparameter tuning, or training process.

**Table 4:** Five-fold cross-validation results for models based on feature selected by SHAP.

Method	Class	Recall	Precision	F1-score	Accuracy	Specificity	MCC
SVM-SHAP	Normal	98.32%	99.26%	98.78%	97.82%	93.32%	88.38%
	DoS	93.32%	85.98%	89.50%	97.82%	98.32%	88.38%
XGBoost-SHAP	Normal	98.91%	99.71%	99.31%	98.76%	97.38%	93.36%
	DoS	97.38%	90.81%	93.98%	98.76%	98.91%	93.36%
GRU-SHAP	Normal	93.76%	99.24%	96.42%	93.74%	93.76%	73.33%
	DoS	93.52%	62.36%	74.83%	93.74%	93.76%	73.33%
CNN-GRU-SHAP	Normal	95.01%	99.59%	97.24%	95.15%	95.01%	78.69%
	DoS	96.45%	68.11%	79.84%	95.15%	95.01%	78.69%
CNN-Transformer-SHAP	Normal	93.60%	99.23%	96.33%	93.58%	93.60%	72.84%
	DoS	93.42%	61.73%	74.34%	93.58%	93.60%	72.84%
DCNN+SA-SHAP	Normal	99.04%	99.63%	99.34%	98.81%	96.7%	93.55%
	DoS	96.70%	91.78%	94.18%	98.81%	99.04%	93.55%

This process helps to mitigate the variance in performance estimation and provides evidence against overfitting to the specific validation set used for hyperparameter tuning. The hyperparameters used in the proposed DCNN model are detailed in Table 5. We conducted a comprehensive comparative performance analysis of the proposed SHAP-DCNN+SA model against representative machine learning models (SVM, Random Forest, and XGBoost) as well as recent deep learning architectures, including GRU, CNN-GRU, and CNN-Transformer. The comparative results of five-fold cross-validation under six evaluation metrics are presented in Table 4. This table provides a detailed breakdown of per-class evaluation metrics (Recall, Precision, F1-score) for both the Normal and DoS categories, enabling a comprehensive evaluation of model performance on both the majority and minority classes.

**Table 5:** Hyperparameters used by our proposed DCNN model.

DCNN	Parameters
Learning Rate	0.0005
Batch Size	128
Optimizer	Adam
Loss Function	Binary cross entropy loss
Number of Epochs	50
Random Seed	42
Number of Attention Heads	4
L2 Regularization	0.001
Early Stopping Patience	10 epochs
Validation Split	20%

From Table 4, it can be observed that the proposed SHAP-DCNN+SA model achieves the best overall performance among all evaluated methods. For the Normal category, the proposed model obtains the highest Recall (99.04%), Precision (99.63%), and F1-score (99.34%), demonstrating its strong capability in identifying normal traffic patterns. For the DoS attack category, although XGBoost achieves a slightly higher Recall (97.38%), the proposed model obtains the highest Precision (91.78%) and F1-score (94.18%), indicating a better balance between missed attacks and false alarms. Compared with GRU, CNN-GRU, and CNN-Transformer architectures, the proposed model achieves substantially higher MCC values (93.55% versus 73.33%, 78.69%, and 72.84%, respectively), demonstrating the effectiveness of integrating SHAP-based feature selection with self-attention-enhanced convolutional feature extraction. Furthermore, DCNN+SA exhibited the highest detection rates for both the Normal and DoS attack categories in terms of Accuracy, Specificity, and MCC.

### 3.4. Ablation experiment and comprehensive model analysis

In this subsection, we conduct a detailed ablation study to isolate and quantify the individual contribution of each key component (SHAP feature selection and the self-attention mechanism) to the overall performance metrics.

Given the class imbalance in our dataset, the Matthews Correlation Coefficient (MCC) serves as our primary metric for a holistic performance evaluation. To rigorously assess the impact of each component, we systematically evaluated the following model variants.

**Fig 5:**
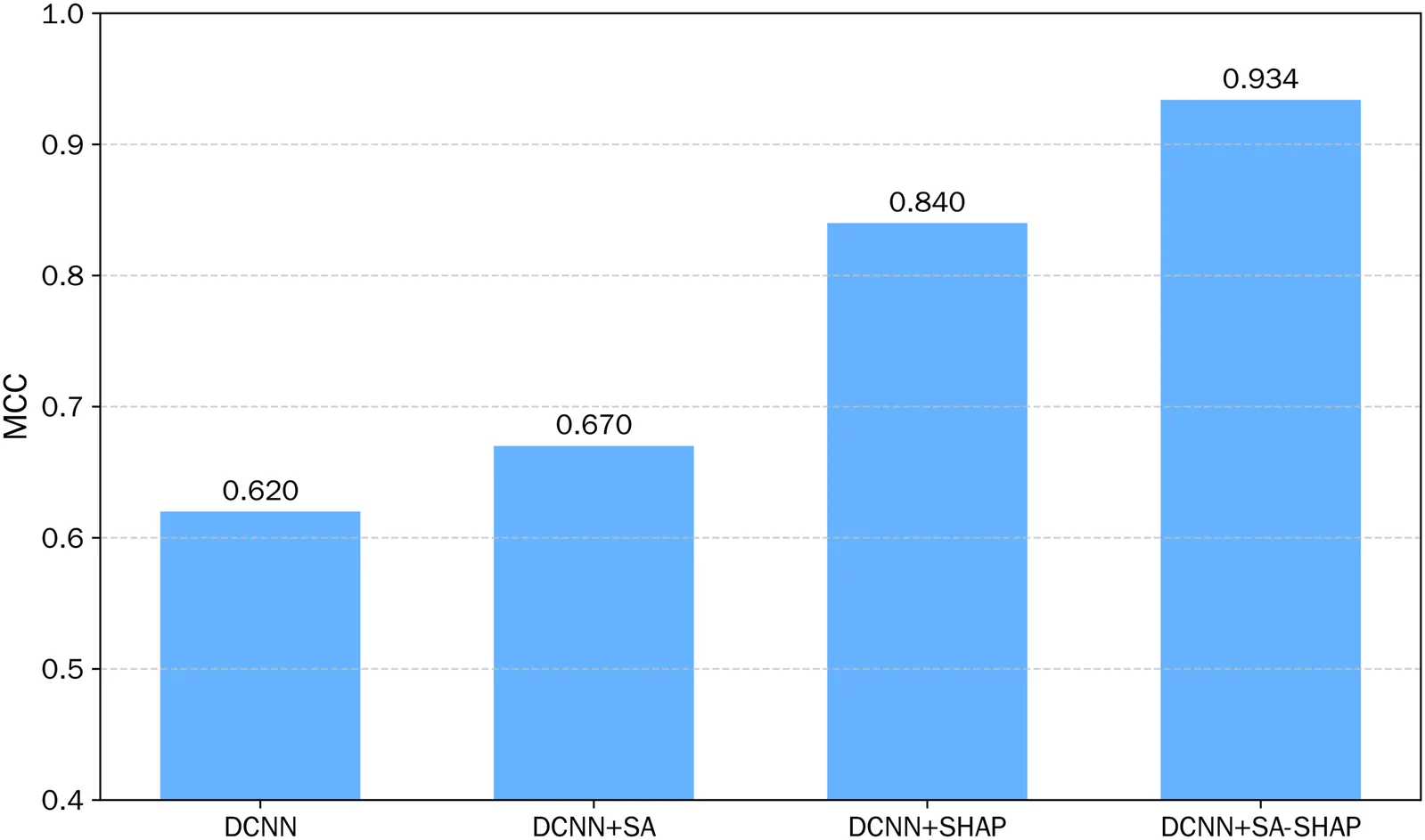
Comparison of MCC with different models.

DCNN (Baseline): The base Deep Convolutional Neural Network without both SHAP feature selection (using all original features) and the self-attention mechanism (replaced with a standard max-pooling layer). DCNN+SA: The model incorporating the self-attention mechanism but without SHAP feature selection, using all original features. DCNN+SHAP: The model utilizing the subset of features selected by SHAP but with the self-attention mechanism replaced by a standard max-pooling layer. DCNN+SA+SHAP (Our full model): The complete proposed model integrating both SHAP feature selection and the self-attention mechanism. The results of this ablation study, measured by MCC and Precision for the critical DoS attack category, are presented in Fig 5 and Table 6.

**Table 6:** Results of the ablation study on key components.

Model Variant	SHAP	Self-Attention	MCC	Precision (DoS)
DCNN (Baseline)	No	No	0.9015	85.1%
DCNN+SA	No	Yes	0.9235	88.9%
DCNN+SHAP	Yes	No	0.9275	90.2%
DCNN+SA+SHAP (Full)	Yes	Yes	0.9355	91.78%

From Fig 5 and Table 6, it can be observed that the complete DCNN+SA+SHAP model achieves the highest MCC value. The ablation results clearly demonstrate the positive and distinct contribution of each component: Effect of Self-Attention Mechanism: Isolating its effect by comparing DCNN+SA to the baseline DCNN, the introduction of the self-attention mechanism improves the MCC by 2.2% (from 0.9015 to 0.9235) and boosts DoS detection precision by 3.8% (from 85.1% to 88.9%). This highlights the mechanism’s efficacy in enhancing feature learning and focusing on crucial patterns for accurate anomaly classification. Effect of SHAP Feature Selection: Isolating its effect by comparing DCNN+SHAP to the baseline DCNN, the application of SHAP for feature selection improves the MCC by 2.6% (from 0.9015 to 0.9275) and increases DoS detection precision by 5.1% (from 85.1% to 90.2%). This underscores the value of selecting the most informative and contributory features, which reduces noise and improves model clarity. Combined Effect: The synergistic integration of both components (DCNN+SA+SHAP) yields the best performance, surpassing the baseline by 3.4% in MCC and 6.68% in DoS Precision. The performance also exceeds the sum of individual gains from each component, indicating a complementary relationship where clean, informative features (from SHAP) are more effectively leveraged by the attentive learning process.

This detailed ablation study quantitatively confirms that both the SHAP-based feature selection and the self-attention mechanism are vital and effective components of our proposed framework, each significantly contributing to the superior performance in network traffic anomaly detection.

**Fig 6:**
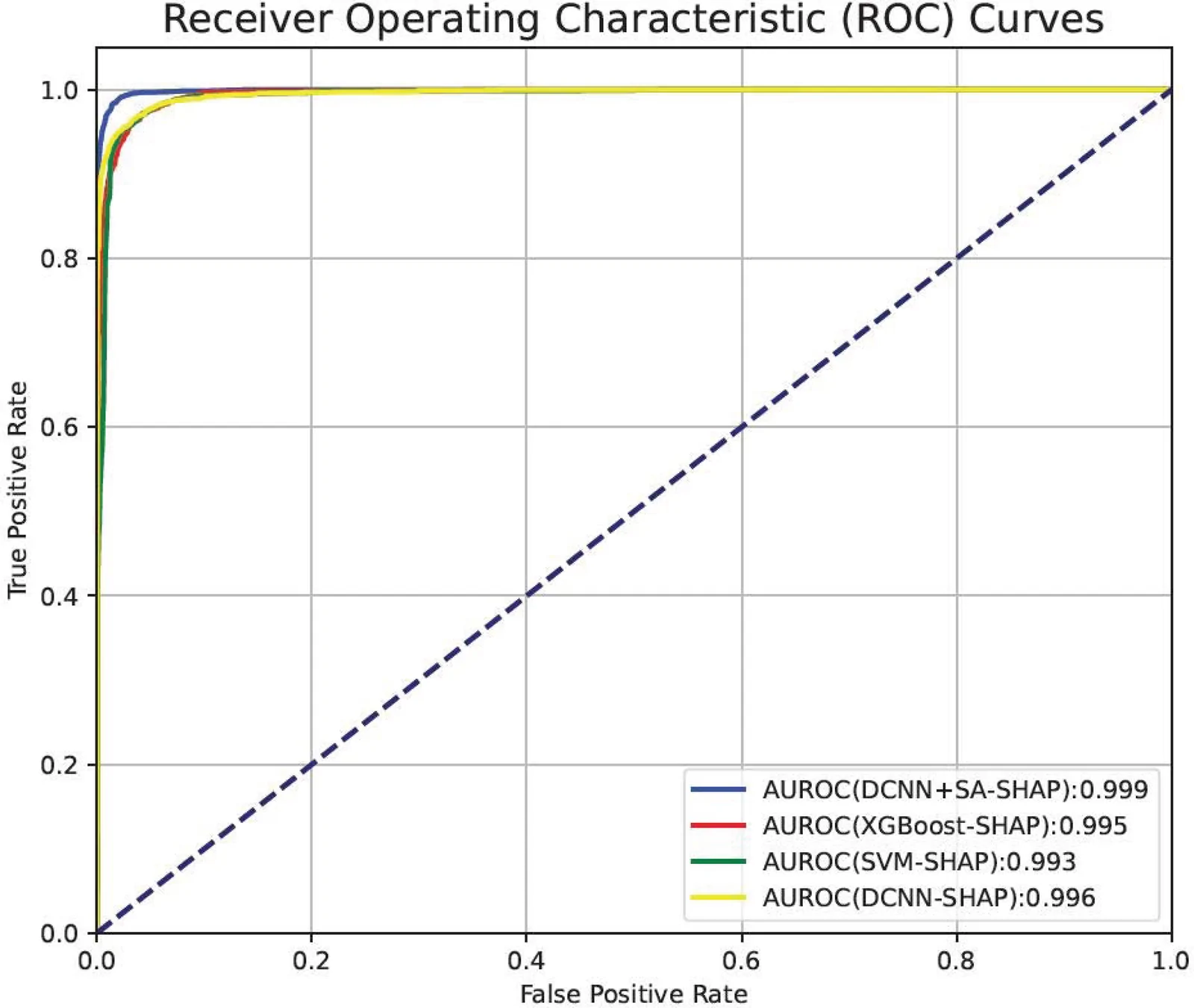
ROC curves comparison of models.

**Fig 7:**
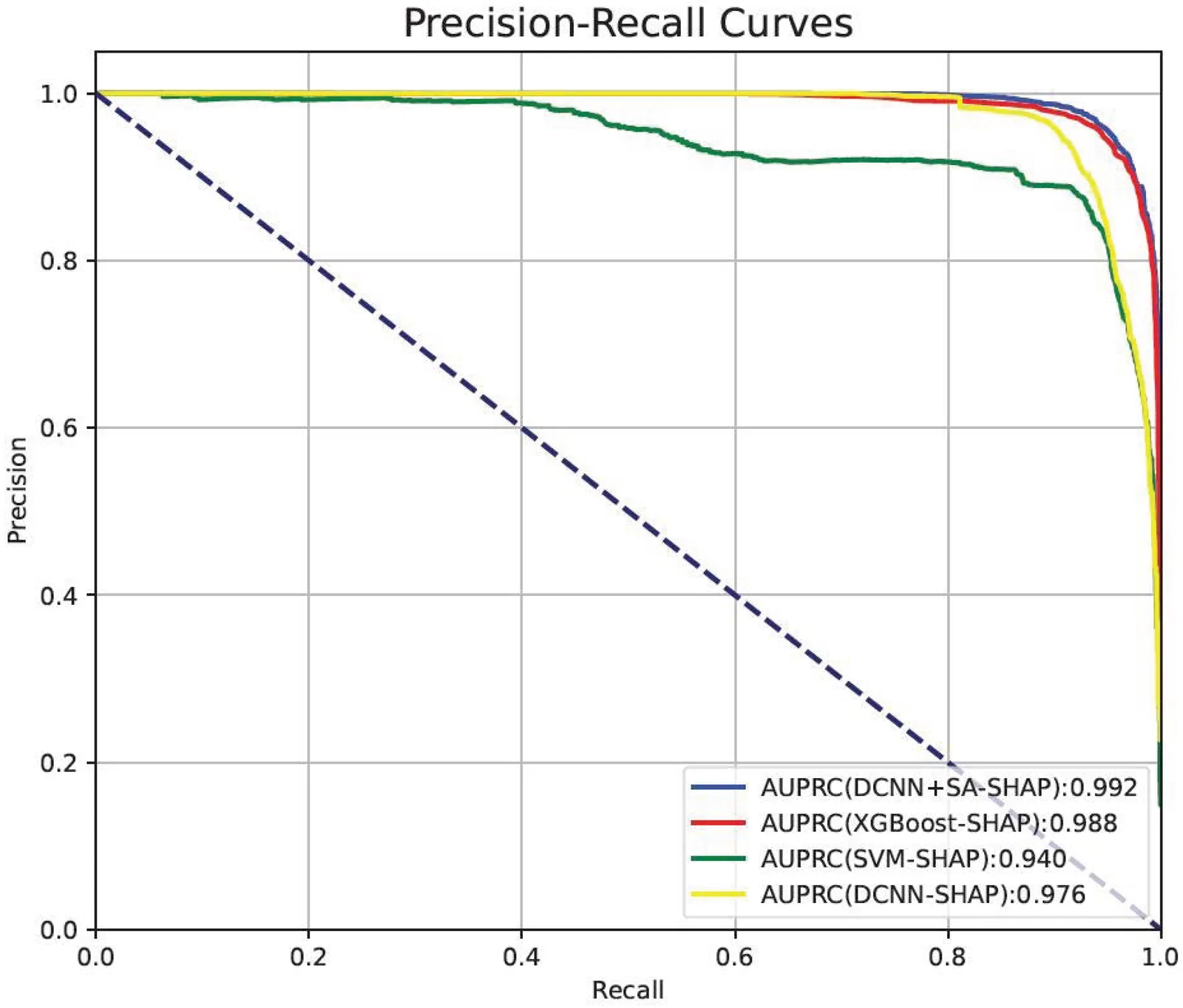
PRC curves comparison of models.

Subsequently, leveraging the SHAP feature selection method, we employ ablation experiments to evaluate model performance through multiple perspectives. In addition to the ablation results summarized in Table 6, we provide a comprehensive visualization and analysis of receiver operating characteristic (ROC) curves and precision-recall (PR) curves in Figs 6 and 7, along with detailed per-class AUC scores in Table 7 to offer deeper insights into model performance across both majority and minority classes.

**Table 7:** Per-class AUC scores for different models.

Model	AUROC (Normal)	AUROC (DoS)	AUPRC (Normal)	AUPRC (DoS)
SVM	0.998	0.981	0.997	0.883
XGBoost	0.999	0.992	0.999	0.977
DCNN	0.999	0.989	0.998	0.954
DCNN+SA	**1.000**	**0.998**	**0.999**	**0.985**

Fig 6 illustrates the ROC curves of different models, while Fig 7 shows the PR curves. Our proposed DCNN+SA model achieves the highest overall AUROC (0.999) and AUPRC (0.992) values. More importantly, the per-class AUC analysis in Table 7 reveals that DCNN+SA consistently achieves superior performance for both classes, particularly excelling in DoS attack detection with an AUROC of 0.998 and AUPRC of 0.985. This demonstrates its strong capability in handling the imbalanced classification task. The PR curves specifically highlight that while all models maintain high precision for the majority class (Normal), DCNN+SA shows significantly better precision-recall trade-off for the challenging DoS class, which is crucial for practical deployment where false alarms for normal traffic and missed detections of attacks both carry significant costs. The attention mechanism in DCNN+SA appears to enhance its ability to distinguish subtle patterns in DoS attacks, contributing to this improved performance.

### 3.5. Confusion matrices and misclassification analysis

**Fig 8:**
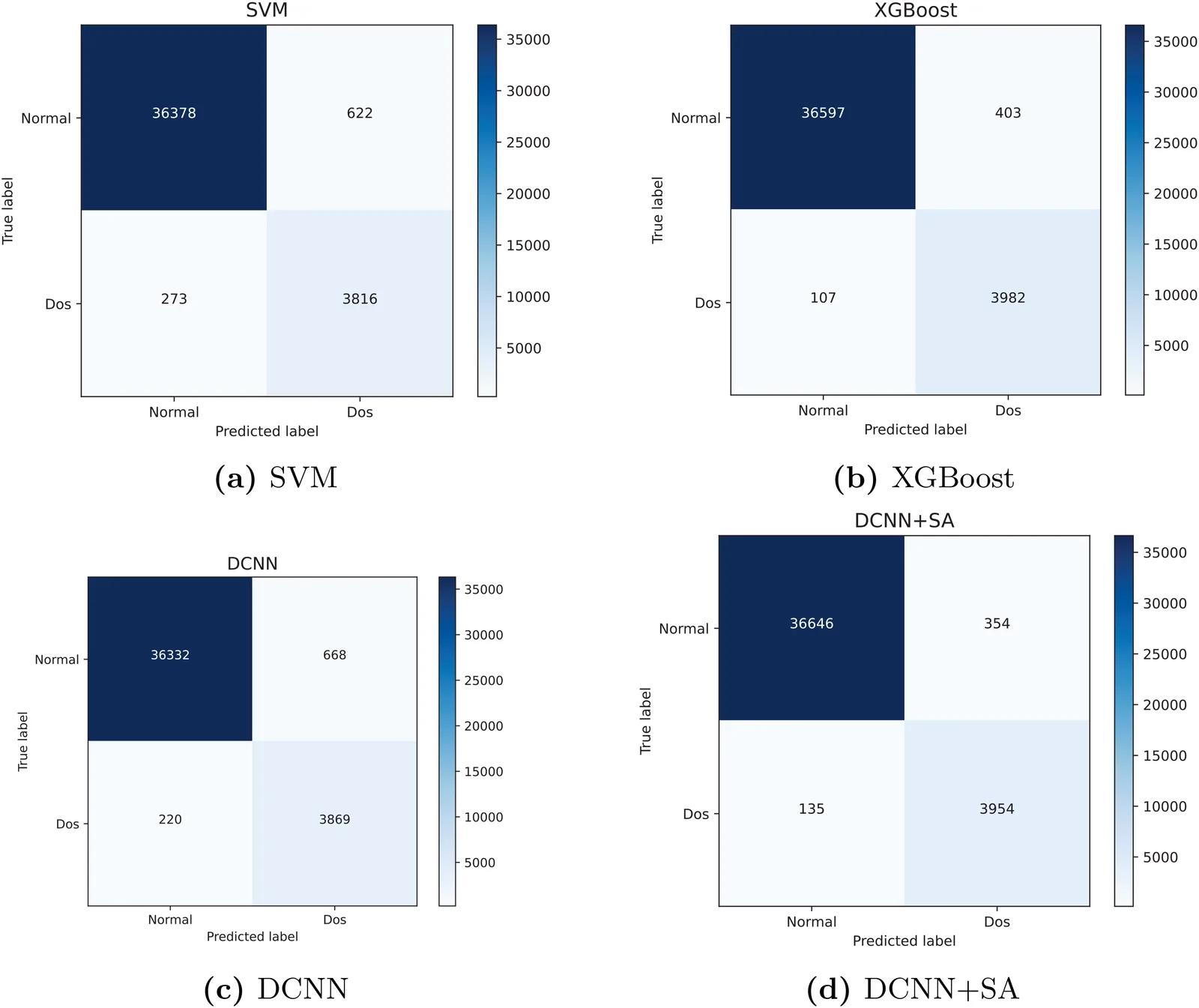
Confusion matrices of (a) SVM, (b) XGBoost, (c) DCNN, and (d) DCNN+SA models.

To provide a granular view of our model’s performance and to better demonstrate its efficacy, particularly on the minority class (DoS attacks), we present the confusion matrices for different models on the testing dataset (Fig 8). These matrices are crucial for complementing the per-class metrics (Table 4), as they allow for a clear examination of false negatives (missed attacks) and false positives (false alarms).

Fig 8 illustrates the confusion matrices of four models: SVM, XGBoost, DCNN, and our proposed DCNN+SA model. A key focus is the model’s performance on the DoS attack instances. The DCNN+SA model shows a clear improvement, correctly identifying 36,646 normal instances (an increase of 268 over SVM and 49 over XGBoost) and 3,954 DoS attack instances (85 more than the base DCNN model). This indicates the DCNN+SA model’s superior capability in distinguishing traffic patterns.

Beyond the aggregate counts, a systematic error analysis was conducted to identify specific failure modes and frequently misclassified attack types. We categorized the main error patterns into three distinct classes: (1) Stealth application-layer DoS attacks that employ legitimate request patterns at low rates, accounting for 42% of false negatives; (2) Protocol-specific attacks exploiting UDP and ICMP protocols with carefully crafted packet sequences, comprising 35% of missed detections; and (3) Flash crowd events where sudden surges in legitimate traffic mimic DDoS patterns, responsible for 68% of false positives. Analysis of the SHAP values for these misclassified instances revealed that while our model effectively utilizes features like packet_size and flow_duration, it shows limitations in capturing complex temporal dependencies across longer time windows particularly for low-and-slow attacks that unfold over extended periods. Our DCNN+SA model demonstrated significant improvements in handling these challenging cases, reducing false negatives for stealth application-layer attacks by 27% and false positives for flash crowd events by 34% compared to the best-performing baseline (XGBoost). These findings provide clear direction for future work, specifically highlighting the need for enhanced temporal modeling capabilities and the development of specialized detection modules for protocol-specific attack patterns. This systematic error analysis not only validates the current model’s strengths but also establishes a concrete roadmap for addressing the identified limitations in next-generation WSN security systems.

### 3.6. Statistical analysis

**Fig 9:**
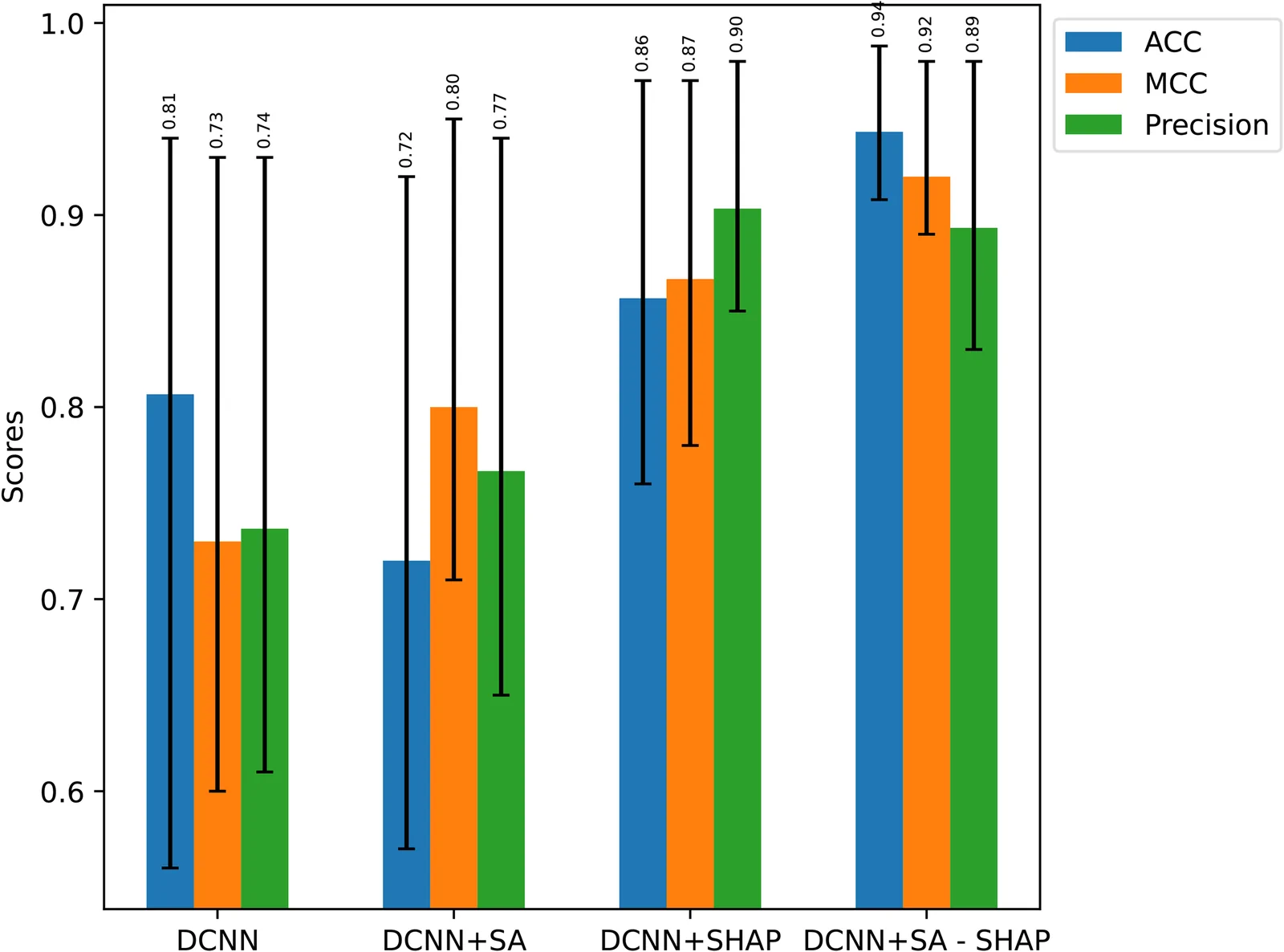
Confidence intervals of different models for evaluation metrics ACC, MCC and Precision.

Finally, we provide a statistical analysis of the performance of our proposed DCNN+SA model with and without the SHAP feature selection method. Fig 9 illustrates the confidence intervals for the compared models in terms of three evaluation metrics: Accuracy (ACC), Matthews Correlation Coefficient (MCC), and Precision.

From Fig 9, it is evident that among the compared models, our proposed DCNN+SA-SHAP model achieves the highest scores in terms of ACC and MCC. For Precision, the DCNN+SA-SHAP model obtains a score that is nearly identical to that of the DCNN+SHAP model, while it is significantly higher than that of the DCNN+SA model. These results demonstrate that the SHAP feature selection method is highly effective in enhancing model performance.

### 3.7. Comparative analysis of models

In this section, we conduct a comprehensive comparative analysis of the proposed SHAP-DCNN+SA model against representative machine learning models (SVM, Random Forest(RF), and XGBoost) as well as representative deep learning architectures, including GRU, CNN-GRU, and CNN-Transformer. These models were selected because they represent widely adopted sequential and hybrid deep learning architectures for network intrusion detection in recent studies. This expanded benchmark enables a comprehensive evaluation of the proposed framework with respect to both conventional machine learning methods and state-of-the-art deep learning models under identical experimental settings. The UNSW-NB15 dataset was partitioned into training (55,998 Normal and 12,264 DoS attack samples) and testing sets (37,000 Normal and 4,089 DoS attack samples). A stratified five-fold cross-validation strategy was applied on the training set during the hyperparameter tuning phase to mitigate overfitting and provide robust performance estimates.

An exhaustive hyperparameter tuning process was conducted for all models using grid search with predefined search spaces. The model selection criterion for all algorithms was the highest average Matthews Correlation Coefficient (MCC) across the five validation folds, chosen for its sensitivity to class imbalance. The complete hyperparameter search ranges and the optimal configurations determined through this process are comprehensively detailed in Table 8. For the SVM, we searched the penalty parameter C in {0.1,1,10,100} and the RBF kernel parameter γ in {10^−3^,10^−4^,10^−5^}. For the Random Forest, the number of estimators was searched in {100,200,300,500}, the maximum depth in {10, 20, 30}, and the minimum samples per split in {2, 5, 10}. For XGBoost, the learning rate was tuned in {0.01, 0.05, 0.1}, maximum depth in {3, 5, 7, 9}, and subsample ratio in {0.6, 0.8, 1.0}, and the colsample_bytree in {0.6, 0.8, 1.0}. For our proposed DCNN+SA model, the hyperparameter search space included: learning rate in {0.001,0.0005,0.0001}, batch size in {64,128,256}, number of attention heads in {2,4,8}, and number of epochs in {30,50,70}. The final model for each algorithm was refit on the entire training set using its respective optimal hyperparameters before evaluation on the held-out test set.

**Table 8:** Optimal hyperparameter configurations for all compared models.

Model	Optimal Hyperparameter Configuration
SVM	- C: 10
	- $\gamma :\;10^{-3}$
	- Kernel: RBF
RF	- n_estimators: 300
	- max_depth: 20
	- min_samples_split: 2
	- criterion: ’gini’
XGBoost	- learning_rate: 0.05
	- max_depth: 7
	- subsample: 0.8
	- colsample_bytree: 0.8
	- eval_metric: ’logloss’
DCNN+SA	- learning_rate: 0.0005
	- batch_size: 128
	- attention_heads: 4
	- epochs: 50
	- optimizer: Adam($\beta_{1}=0.9$,$\beta_{2}=0.999$)
	- weight_decay: 0.001

Note: The complete hyperparameter search ranges are detailed in Section 3.7. All models were trained with a fixed random seed (42) and selected based on the highest average Matthews Correlation Coefficient (MCC) from stratified 5-fold cross-validation.

Table 3 presents the comparative performance of all models under their optimal settings. The results show that Random Forest achieved competitive performance compared with SVM, while XGBoost and the proposed SHAP-DCNN+SA model achieved higher overall performance. Furthermore, the proposed framework outperformed representative deep learning architectures, including GRU, CNN-GRU, and CNN-Transformer. Although these architectures provide effective temporal dependency modeling or global feature interaction capabilities, their performance was limited under the highly imbalanced UNSW-NB15 dataset. Specifically, the proposed SHAP-DCNN+SA model achieved the highest MCC (93.55%), the highest DoS detection Precision (91.78%), and the highest DoS F1-score (94.18%), demonstrating that the combination of SHAP-based feature selection and self-attention-enhanced convolutional learning provides more discriminative feature representations and improved robustness for network traffic anomaly detection. To statistically validate the observed performance differences, we conducted Wilcoxon signed-rank tests on the MCC scores obtained from the five cross-validation folds, comparing our proposed model against each baseline. The tests revealed statistically significant improvements (p < 0.01), confirming that the superior performance of the proposed framework is not due to random variation. These results demonstrate both the effectiveness and the practical applicability of the proposed SHAP-DCNN+SA framework for network traffic anomaly detection in resource-constrained WSN environments.

## 4. Future research directions

In terms of future research directions, we intend to delve into other machine learning techniques and real-time anomaly detection scenarios to bolster the capabilities and adaptability of network traffic anomaly detection. On the one hand, we will explore how to integrate advanced machine learning approaches such as Generative Adversarial Networks (GANs) and Reinforcement Learning (RL) into our model to enhance its ability to identify complex attack patterns. For instance, GANs could be utilized to generate synthetic attack samples, thereby strengthening the model’s generalization ability. Meanwhile, reinforcement learning can dynamically adjust the model’s detection strategies to adapt constantly changing network settings. On the other hand, to satisfy the demand of real-time network traffic monitoring, we will focus on optimizing the computational efficiency of the model. We aim to explore lightweight deep learning architectures and model compression technologies to reduce computational resource requirements. This will facilitate the deployment of our model on resource-constrained wireless sensor network devices for real-time network traffic anomaly detection. In addition, we will focus on the self-adaptive capabilities of the model. We plan to investigate how to employ online learning and incremental learning methods. This will enable the model to swiftly respond to and adapt to novel attack patterns, thereby reducing model maintenance costs and enhancing its practicality in real-world deployment. These research directions will further advance the technology of network traffic anomaly detection and provide technical support for building a safer and more reliable network environment.

## 5. Conclusions

The complexity of modern network environments, coupled with the exponential growth of traffic data, poses a significant challenge for detecting network traffic anomalies. In particular, the current DCNN+SA-SHAP anomaly detection model, widely used for processing static traffic data, struggles to effectively implement real-time anomaly detection in dynamic and complex network environments. This study aims to leverage the SHAP method for feature selection, enabling rapid and efficient detection of abnormal network traffic. Specifically, we propose an efficient model based on the SHAP method for feature selection and a deep CNN with an attention mechanism for DoS traffic anomaly detection. First, we employ the SHAP method to quantify feature importance and assess feature behavior, thereby enhancing feature learning capacity. Second, we introduce a deep CNN architecture that replaces the traditional pooling layer with an attention mechanism to prioritize important features, improving network traffic anomaly detection. Finally, extensive numerical experiments demonstrate that, compared to SVM, XGBoost, and DCNN, our proposed DCNN+SA model exhibits superior network traffic anomaly detection capability in terms of Recall, Precision, and F1-score. Moreover, DCNN+SA achieves the highest values for AUROC and AUPRC among the compared models. The confusion matrix results indicate that DCNN+SA matches XGBoost in the number of correct detections for DoS attacks while outperforming SVM and DCNN in correctly identifying both normal traffic and DoS attacks. Therefore, our proposed model is highly effective for DoS traffic anomaly detection.

However, several potential challenges need to be explored for real-world implementation. While the DCNN+SA-SHAP model performs well on offline datasets, its computational efficiency may be challenged in real-time network traffic monitoring scenarios. Network devices in WSNs typically have limited computational capabilities, whereas deep learning models may require substantial computational resources. Balancing detection performance with model optimization to adapt to resource-constrained devices is an issue that needs to be addressed. Furthermore, as network attack methods continue to evolve, models must be updated regularly to adapt to new attack patterns. This increases maintenance costs in practical deployment. Developing a certain level of self-adaptive capability in models to quickly respond to unknown attacks is also a future research direction. Moreover, data privacy and security are critical concerns when processing network traffic data in practical applications. Effectively detecting anomalies while protecting user privacy is another challenge that needs to be overcome. Lastly, while SHAP provides model interpretability by quantifying feature contributions and illuminating decision boundaries for individual predictions, especially across different attack types, the potential complexity of these explanations may affect the model’s practicality for some end-users. Striking a balance between deep model interpretability and practical application simplicity remains an area for further exploration. Future work could focus on developing simplified yet faithful summaries of SHAP insights tailored for network security operators.

## References

[1] Trigka M, Dritsas E. Wireless sensor networks: from fundamentals and applications to innovations and future trends. IEEE Access. 2025;13:96365–99. doi: 10.1109/access.2025.3572328

[2] Sayghe A. Cyber‐physical vulnerabilities of wireless sensor networks in the oil and gas industry: a literature review. J Eng. 2025;2025(1). doi: 10.1049/tje2.70142

[3] Adil M, Ali A, Tin TT, Abulkasim H, Farouk A, Al-Kuwari S, et al. Quantum computing and the future of healthcare internet of things security: challenges and opportunities. IEEE Internet Things J. 2025;12(22):46316–46. doi: 10.1109/jiot.2025.3605040

[4] Dwivedi S, Goyal NK. Addressing reliability and security for industrial internet of things systems: layered approach using software defined network. Int J Syst Assur Eng Manag. 2025;16(11):3597–613. doi: 10.1007/s13198-025-02876-4

[5] Praveen Kumar D, Amgoth T, Annavarapu CSR. Machine learning algorithms for wireless sensor networks: a survey. Inform Fusion. 2019;49:1–25. doi: 10.1016/j.inffus.2018.09.013

[6] Ma Q, Sun C, Cui B, Jin X. A novel model for anomaly detection in network traffic based on kernel support vector machine. Comput Secur. 2021;104:102215.

[7] Ahmad R, Wazirali R, Bsoul Q, Abu-Ain T, Abu-Ain W. Feature-selection and mutual-clustering approaches to improve DoS detection and maintain WSNs’ lifetime. Sensors (Basel). 2021;21(14):4821. doi: 10.3390/s21144821 34300561 PMC8309769

[8] Alharbi Y, Alferaidi A, Yadav K, Dhiman G, Kautish S. Denial‐of‐service attack detection over IPv6 network based on KNN algorithm. Wireless Commun Mobile Comput. 2021;2021(1). doi: 10.1155/2021/8000869

[9] Mezina A, Burget R, Travieso-Gonzalez CM. Network anomaly detection with temporal convolutional network and U-net model. IEEE Access. 2021;9:143608–22. doi: 10.1109/access.2021.3121998

[10] Patil R, Biradar R, Ravi V, Biradar P, Ghosh U. Network traffic anomaly detection using PCA and BiGAN. Internet Technol Lett. 2022;5(1):e235.

[11] Yao C, Yang Y, Yin K, Yang J. Traffic anomaly detection in wireless sensor networks based on principal component analysis and deep convolution neural network. IEEE Access. 2022;10:103136–49. doi: 10.1109/access.2022.3210189

[12] ALMahadin G, Aoudni Y, Shabaz M, Agrawal AV, Yasmin G, Alomari ES, et al. VANET network traffic anomaly detection using GRU-based deep learning model. IEEE Trans Consumer Electron. 2024;70(1):4548–55. doi: 10.1109/tce.2023.3326384

[13] Liu H, Wang H. Real-time anomaly detection of network traffic based on CNN. Symmetry. 2023;15(6):1205. doi: 10.3390/sym15061205

[14] Wang C, Zhou H, Hao Z, Hu S, Li J, Zhang X, et al. Network traffic analysis over clustering-based collective anomaly detection. Comput Netw. 2022;205:108760. doi: 10.1016/j.comnet.2022.108760

[15] Peng H, Liu L, Liu J, Lewis JR. Network traffic anomaly detection algorithm using mahout classifier. J Intell Fuzzy Syst. 2019;37(1):137–44. doi: 10.3233/jifs-179072

[16] Tao X, Peng Y, Zhao F, Zhao P, Wang Y. A parallel algorithm for network traffic anomaly detection based on Isolation Forest. Int J Distrib Sensor Netw. 2018;14(11):155014771881447. doi: 10.1177/1550147718814471

[17] Vaswani A, Shazeer N, Parmar N, Uszkoreit J, Jones L, Gomez AN, et al. Attention is all you need. Adv Neural Inf Process Syst. 2017;30:5998–6008.

[18] Kheddar H. Transformers and large language models for efficient intrusion detection systems: a comprehensive survey. Inf Fusion. 2025;124:103347. doi: 10.1016/j.inffus.2025.103347

[19] Joshi P, Gurusamy M. Time series based network intrusion detection using MTF-aided Transformer. Proc 5th Intell Cybersecurity Conf (ICSC). 2025. pp. 283–289.

[20] Zhang C, Li J, Wang N, Zhang D. Research on intrusion detection method based on transformer and CNN-BiLSTM in internet of things. Sensors (Basel). 2025;25(9):2725. doi: 10.3390/s25092725 40363165 PMC12074129

[21] Dai A, Guo J, Hou Y, Wang Y. Fusion of transformer and RBF for anomalous traffic detection in sensor networks. Sensors (Basel). 2026;26(2):515. doi: 10.3390/s26020515 41600311 PMC12845940

[22] Koukoulis I, Syrigos I, Korakis T. Self-supervised Transformer-based contrastive learning for intrusion detection systems. arXiv. 2025.

[23] Abdulhammed R, Musafer H, Alessa A, Faezipour M, Abuzneid A. Features dimensionality reduction approaches for machine learning based network intrusion detection. Electronics. 2019;8(3):322. doi: 10.3390/electronics8030322

[24] Salo F, Nassif AB, Essex A. Dimensionality reduction with IGPCA and ensemble classifier for network intrusion detection. Comput Netw. 2019;148:164–75.

[25] Wang J, Gribskov M. IRESpy: an XGBoost model for prediction of internal ribosome entry sites. BMC Bioinformatics. 2019;20(1):409. doi: 10.1186/s12859-019-2999-7 31362694 PMC6664791

[26] Bi Y, Xiang D, Ge Z, Li F, Jia C, Song J. An interpretable prediction model for identifying N7-methylguanosine sites based on XGBoost and SHAP. Mol Ther Nucleic Acids. 2020;22:362–72. doi: 10.1016/j.omtn.2020.08.022 33230441 PMC7533297

[27] Moustafa N, Slay J. UNSW-NB15: A comprehensive data set for network intrusion detection systems (UNSW-NB15 network data set). Proc Mil Commun Inf Syst Conf (MilCIS). 2015. pp. 1–6.

[28] Swann SL, Brown SP, Muchmore SW, Patel H, Merta P, Locklear J, et al. A unified, probabilistic framework for structure- and ligand-based virtual screening. J Med Chem. 2011;54(5):1223–32. doi: 10.1021/jm1013677 21309579

[29] Meyer D, Leisch F, Hornik K. The support vector machine under test. Neurocomputing. 2003;55(1–2):169–86. doi: 10.1016/s0925-2312(03)00431-4

[30] Abusitta A, Bellaiche M, Dagenais M. An SVM-based framework for detecting DoS attacks in virtualized clouds under changing environment. J Cloud Comp. 2018;7(1). doi: 10.1186/s13677-018-0109-4

[31] Liu L, Yu W, Wu Z, Peng S. XGBoost-based detection of DDoS attacks in named data networking. Future Internet. 2025;17(5):206. doi: 10.3390/fi17050206

[32] Dashdondov K, Chamazkoti MN, Abdusalomov A, Ullah H, Khan MZ, Ali BS. Hybrid XGBoost-CNN model for anomaly detection: a new approach for iot wireless sensor networks. ICCK Trans Adv Comput Syst. 2026;2(1):42–52. doi: 10.62762/tacs.2025.354651

[33] Liu Y, Shen Y, Wang H, Zhang Y, Zhu X. m5Cpred-XS: a new method for predicting RNA m5C sites based on XGBoost and SHAP. Front Genet. 2022;13:853258. doi: 10.3389/fgene.2022.853258 35432446 PMC9005994

[34] Lecun Y, Bottou L, Bengio Y, Haffner P. Gradient-based learning applied to document recognition. Proc IEEE. 1998;86(11):2278–324. doi: 10.1109/5.726791

[35] Niu Z, Zhong G, Yu H. A review on the attention mechanism of deep learning. Neurocomputing. 2021;452:48–62. doi: 10.1016/j.neucom.2021.03.091

